# Response to major peanut and peach allergens in a population of children allergic to peanut

**DOI:** 10.1186/2045-7022-4-S2-P29

**Published:** 2014-03-17

**Authors:** Miguel Blanca

**Affiliations:** 1 Allergy Service (Hospital Civil), Plaza Hospital Civil (s/n), Malaga, 29009, Spain

## 

Response to major peanut and peach allergens in a population of children allergic to peanut. Garcia-Blanca A1, Gomez F2, Aranda A3, Blanca-Lopez N1, Mayorga C3, Torres2 MJ, Diaz-Perales A4, Blanca M2, Canto G1. 3Research Laboratory, Carlos Haya Hospital-FIMABIS, Málaga, Spain, 2Allergy Service, Carlos Haya Hospital, Málaga, Spain, 4Plant Biotechnology Institute (UPM-INIA), Madrid, Spain,1Allergy Service, Infanta Leonor Hospital, Madrid, Spain.

## Background

We studied sensitization to peanut allergens in a population of children allergic to food of vegetal origin as well as the relationship with peach allergy.

## Methods

All children referring symptoms to allergens of vegetal origin were included. They were classified in two groups: A) those allergic to peanut with good tolerance to peach and B) those allergic to peach and peanut. The IgE response to several peanut and peach allergens and the relationship with the different clinical entities was analyzed.

## Results

A total of 270 children evaluated, the 24% referred symptoms after peanut ingestion. Mean age was 6.5 y o with 63% sensitized to pollens. Most relevant pollens involved were grass, olive, plantain and artemisia. From the total group 33% had anaphylaxis, 40% had urticaria and 21 OAS. IgE positive was found to peanut allergens (Ara h 1,2,3 9) and to Pru p 3 isolated or in combinations being Ara h 2 the most relevant found. When we compared the groups with symptoms only to peanut with those who developed symptoms to both there were differences in the sex ratio (p 0.02), the atopic status (p 0,05). More anaphylaxis in those of group A and more OAS in those of group B was found. When we analyzed the IgE response to the individual allergens the pattern of response was similar in both groups.

## Conclusions

Although peanut allergy is frequent in a children population the pattern of response and the relationship with peach allergy is complex. A tendency to see more anaphylaxis in children only sensitized to peanut and in OAS in those sensitized to peanut and peach was observed. These data require more detailed studies on the individual response to isolated allergens in order to establish the individual response and the cross-reactivity between both families of allergens.

